# Directional Torsion Sensor Based on a Two-Core Fiber with a Helical Structure

**DOI:** 10.3390/s23062874

**Published:** 2023-03-07

**Authors:** Zhuo Song, Yichun Li, Junhui Hu

**Affiliations:** 1 Guangxi Key Laboratory of Nuclear Physics and Technology, College of Physics Science and Technology, Guangxi Normal University, Guilin 541004, China; 2 Key Laboratory of Specialty Fiber Optics and Optical Access Networks, Joint International Research Laboratory of Specialty Fiber Optics and Advanced Communication, Shanghai Institute for Advanced Communication and Data Science, Shanghai University, Shanghai 200444, China

**Keywords:** fiber-optic torsion sensor, helical two-core fiber, Mach–Zehnder interferometer

## Abstract

A fiber-optic torsion sensor based on a helical two-core fiber (HTCF) is proposed and experimentally demonstrated for simultaneously measuring torsion angle and torsion direction. The sensor consists of a segment of HTCF and two single-mode fibers (SMFs) forming a Mach–Zehnder interferometer (MZI). The helical structure is implemented by pre-twisting a 1 cm long two-core fiber (TCF). The performance of the sensor with pre-twisted angles of 180°, 360°, and 540° is experimentally analyzed. The results show that the sensor can realize the angular measurement and effectively distinguish the torsion direction. It is worth noting that the sensor has maximum sensitivity when the pre-twist angle is 180 degrees. The obtained wavelength sensitivities of torsion and temperature are 0.242 nm/(rad/m) and 32 pm/°C, respectively. The sensor has the advantages of easy fabrication, low cost, compact structure, and high sensitivity, which is expected to yield potential applications in fields where both torsion angle and direction measurements are required.

## 1. Introduction

In recent years, optical fiber torsion sensors have attracted the attention of various scholars. They have been widely used in the fields of aerospace engineering [1,2], geological health monitoring [3], and anthropomorphic robots [4,5] due to the merits of low cost, easy fabrication, and immunity to electromagnetic interference. Nowadays, diversiform types of torsion sensors have been reported; in general, these can be classed into two main categories: optical fiber grating-based torsion sensors and optical fiber interferometer-based torsion sensors.

The grating-based torsion sensor is a typical optical fiber torsion sensor that has undergone significant development recently. Zhang et al. proposed a two-helical long-period fiber gratings (LFBG) torsion sensor. The sensor achieved a sensitivity of 0.115 nm·m/rad in an effective torsion rate range from −5π/2 rad/m to 5π/2 rad/m [6]. Deng et al. presented a pre-twisted LFBG in single-mode fiber with a sensitivity of 0.54 nm/(rad/m) [7]. Similarly, an angle-chirped long-period fiber grating (ACLPFG), which is inscribed in a single-mode fiber via CO_2_ laser, exhibits ~16-fold higher sensitivity than that of the normal long-period fiber grating (LPFG) and its temperature crosstalk is only ~1/10 compared to the normal LPFG [8]. In 2019 and 2020, a double-clad chiral long-period fiber grating (CLPG) [9] and a phase-shifted LFBG [10] were proposed, with a maximum sensitivity of 0.4 nm/(rad/m) and 0.114 nm/(rad/m), respectively. In 2021, Oliveira et al. demonstrated the ability to measure shear strain and torsion loads by bonding an optical fiber to a 3D-printed periodic grooved plate. The achieved sensitivities for the dip transmission ratio were a function of the load of 0.12/mε and 0.21/°, for shear strain and torsion loads ranging from 0∼8 mε and 1∼4 deg, respectively [11]. However, most grating-based torsion sensors use special grating, which requires a complex fabrication process and expensive manufacturing equipment, such as a CO_2_ laser or a femtosecond laser.

On the other hand, the torsion sensors based on optical fiber interferometers mainly depend on the change in optical fiber refractive index or birefringence caused by torsion, which affects the interference spectrum [12,13,14]. However, this type of torsion sensor suffers from the difficulty that the torsion direction cannot be identified. To solve this problem, interferometers with helical or antisymmetric structures have been proposed. Plenty of the work emerging is based on helical twisted photonic crystal fiber (PCF) with quality sensitivity and identifiable direction [15,16,17]. The process of PCF production is complex. The percentage of passing products is low, which is not conducive to the commercial production of sensors. An intensity-modulated directional torsion sensor based on a helical taper fiber is proposed and experimentally demonstrated [18]. Moreover, the sensitivity of the sensor can be improved by using a spiral structure and asymmetric structure. Jiang et al. reported a torsion sensor sandwiching a fraction of dual-side hole fiber (DSHF) between two short multimode fibers (MMFs). The sensor displayed a sensitivity of 1.666 nm/(rad/m) and −1.413 nm/(rad/m) in different rotating directions [19]. Likewise, Xu et al. proposed a novel torsion sensor based on pre-twisted taper DSHF. The results show that the torsion sensitivity of the interferometer was as high as −1.67 nm/(rad·m^−1^) and 1.65 nm/(rad·m^−1^) in the clockwise (CW) and counterclockwise (CCW) rotations, respectively [20].

In this paper, we experimentally demonstrated a highly sensitive torsion sensor based on a HTCF MZI. The HTCF is fabricated by pulling and pre-twisting the two-core fiber. Then, the two ends of the HTCF are fusion-spliced to the two SMFs to form a Mach–Zehnder interferometer. The sensor has the ability to measure the magnitude and direction of the torsion simultaneously. Compared to the aforementioned sensing structures, the sensor offers several merits, such as good mechanical robustness structure and compact size, which further benefits its practical sensing applications.

## 2. Sensor Fabrication

**Figure 1:**
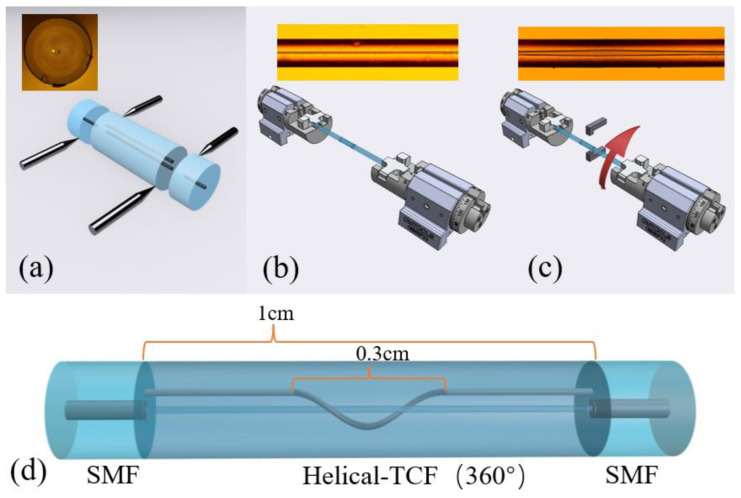
The production process for the sensing structure. (**a**) TCF is subjected to fusion spliced with SMF. (**b**) Keep fiber straight. (**c**) Twist and heat the TCF. (**d**) Schematic diagram of sensing structure (360°).

The schematic of the proposed sensor is shown in Figure 1. The sensor is a Mach–Zehnder interferometer, which is formed by fusion splicing the two ends of the HTCF to the two sections of SMFs. Figure 1a reveals the cross-section image of the TCF. The diameters of the two cores are 6 μm, respectively, located in the center and off-center side of the cladding with a spacing distance of about 10 μm. The cladding diameter of TCF is 125 μm, the same as that of SMF. It is noted that the TCF has a non-coaxial symmetric structure, which is more beneficial to the measurement of torsion angle and direction.

The fabrication process of the sensing structure is as follows: (1) a section of the TCF is subjected to fusion splicing with two pieces of SMF to form the SMF-TCF-SMF structure. During fusion, the length of TCF is strictly controlled at 1 cm under the monitoring of a caliper. (2) Mount the two ends of the SMF-TCF-SMF structure on the rotatable fixture and apply slight stress to the structure to ensure that the fiber is not bent. (3) Light the oxy-hydrogen flame nozzle; the temperature of the oxy-hydrogen flame is about 1800 degrees Celsius. Move the oxy-hydrogen flame nozzle to the central part of the welded structure to make the flame completely envelop the optical fiber. (4) During the heating process, rotate the clamp at different angles (180°, 360°, 540°) clockwise at the same time. The whole heating process lasts for about 20 s. Since the clamp and the vertical axis of the fiber are in the same position, a clockwise twist in the molten state will only change the position and shape of the side core and not the middle core. (5) Wait for the end of the heating process, and the optical fiber will cool down to form a helical structure (HS). 

The view photographs of TCF (left) and the HTCF (right) are disclosed in Figure 1b,c. The left and right graphs correspond to without and with HS, respectively. From Figure 1c, it can be observed that the helical deformation is only introduced into the side cores of TCF, while the SMF-HTCF-SMF structure is still kept straight as a whole. The length of the HS is about 0.3 cm.

**Figure 2:**
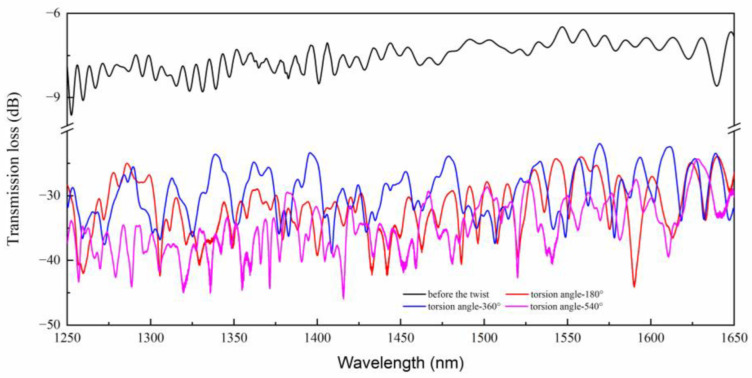
Measured transmission spectra of the MZI without and with HS structure.

The measured transmission spectra of the SMF-TCF-SMF structure and the SMF-HTCF-SMF structure is presented in Figure 2. The black solid line depicts the spectrum before pre-twist, and other solid lines are the spectra after pre-twist with 180°, 360°, and 540° angles. It can be ascertained that by introducing the HS structure, the interference pattern is more obvious and the extinction ratio (ER) of the dips is significantly enhanced. The ER increased from 1 dB to 15 dB, which illustrated that the HTCF could stimulate the cladding mode with a stronger capacity. ER is the transmission loss of the crest minus the trough of a complete waveform. In addition, the introduction of the pre-twisted structure improves the free spectral range (FSR), which improves the measurement range of torsion. The FSR is the crest of a complete waveform minus the spectral width of the crest and it is the spectral interference period.

**Figure 3:**
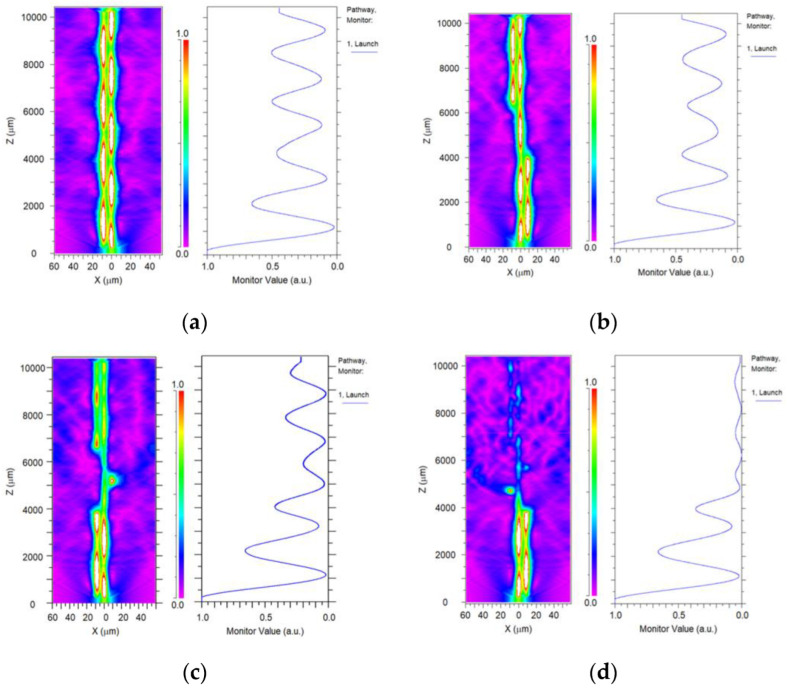
Simulation of power output under different torsion angles: (**a**) 0° (**b**) 180° (**c**) 360° (**d**) 540°.

In order to evaluate the influence of the pre-twist angle on the interference spectrum, the electric field distributions of the SMF-HTCF-SMF structure with 180°, 360°, and 540° angles pre-twist angle of TCF are respectively simulated by using the beam propagation method (BPM). The results are displayed in Figure 3. It can be seen that the side core mode and cladding modes are excited concomitantly by the increase in the pre-twist angle. Besides, with the incremental angle, the light leaks from the fiber core into the cladding more easily, resulting in a decrease in the light energy output.

## 3. Sensing Principle

**Figure 4:**
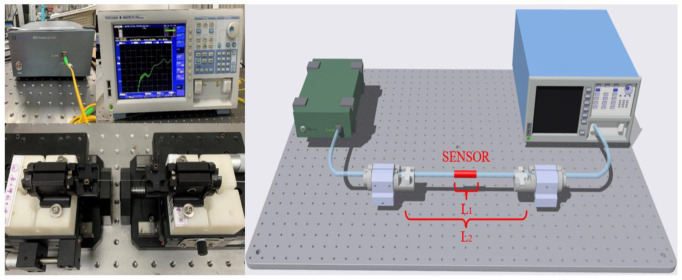
The real product and schematic diagram of the sensing system.

The schematic diagram of the experimental setup is presented in Figure 4. The light travels along the lead-in SMF from the broadband source (BBS) (GM8035) and is then injected into the sensing structure. The central fiber core mode (CCM), side fiber core mode (SCM), and cladding mode (CM) are excited in the HTCF owing to the mode field mismatch of the HTCF and the SMF. The lights from the HTCF are then coupled into the lead-out SMF. Interference occurs on the lead-out SMF due to the optical path difference (OPD). The interference spectra are recorded by an optical spectrum analyzer (OSA). Although multiple cladding modes may be excited, it can be assumed that only one domain cladding mode interferes with the core modes. When the light propagates through the sensing structure, interference among the CCM, the CM, and OCM will arise due to the OPDs. Assuming that the interference is involved among OCM, CCM, and only one CM, the interference intensity generated by N different modes can be expressed by the formula [21,22]:

(1) $$I=\sum_{i=1}^{N}I_{i}+2\sum_{i=1}^{N}\sum_{j=2}^{N}\sqrt{I_{i}I_{j}}\cos(\Delta \varphi_{ij})$$

where *I_i_* and *I_j_* present the intensities of the *i*-th and *j*-th modes. Both *i* and *j* are integers and *j* > *i*. $\Delta \varphi_{ij}$ denotes the phase difference between the two modes. When the phase difference is $\Delta \varphi_{ij}=(2n+1)\pi ,\;n=0,1,2,3\ldots$, the transmission dips occur at:

(2) $$\lambda_{ij}=\frac{2\left[\Delta n_{eff}^{i-j,s}L_{s,TCF}+\Delta n_{eff}^{i-j,h}L_{h,TCF}\right]}{2n+1}$$

The stress is generated in the pre-twisted region (HS region) when the pre-twist is applied to TCF, but the stress in the central fiber core is zero, while the outer edge increasing as a quadratic function [23]. Stressed regions of the TCF undergo refractive index changes, which are proportional to local stress and the material’s elastic–optic coefficients. Therefore, the RI of the pre-twisted TCF is expressed by the formula [24,25]:

(3) $$n(\tau )\approx n_{0}(1+\frac{d^{2}\tau^{2}}{2})$$

where *τ* is the torsion rate (TR), *d* is the radial distance to the center of the fiber, and *n*_0_ is the RI for the untwisted state. In the HS region, since a pre-twisted rate *τ*_0_ has been pre-induced into the fiber and caused RI change, the corresponding RI changes with the applied torsion *τ* can be written as:

(4) $$n^{h}(\tau )\approx n_{0}(1+\frac{d^{2}{\left(\tau \pm \tau_{0}\right)}^{2}}{2})$$

where “+” and “−” mean the applied pre-twist is in the CW and CCW directions, respectively. Since the influence of the twisting process on the length of the non-helical structure is slight, the term $\partial L_{s,TCF}/\partial \tau \;$can be approximately ignored. According to Equation (2), the wavelength shift caused by the applied torsion variation Δ*τ* can be approximately expressed as [19]:

(5) $$\Delta \lambda_{ij}^{\tau }\approx \frac{2}{2n+1}\left[\frac{\partial \Delta n_{eff}^{i-j,s}}{\partial \tau }L_{s,TCF}+\frac{\partial \Delta n_{eff}^{i-j,h}}{\partial \tau }L_{h,TCF}+\Delta n_{eff}^{i-j,h}\frac{\partial L_{h,TCF}}{\partial \tau }\right]\cdot \Delta \tau$$

Moreover, in the proposed sensor with HS, $L_{h,TCF}$ can be calculated from the parametric equations of a cylinder-shaped helical line [19]:

(6) $$\{\begin{array}{cc} \begin{array}{c} x=R\cos\theta \\ y=R\sin\theta \\ z=v\theta \end{array}, & 0 \end{array}<\theta <2k\pi$$

where *x*, *y*, and *z* are the three axes in classical Cartesian coordinates, *R* is the bottom radius, *θ* is the rotation angle of the *R* away from the *x*, *e* indicates the pitch, *h* is the height of the helical structure, *v *=* e*/*2π* is the growth rate of the helical line along the *z*, and *k *=* h*/*e*.

Then, the helical length can be obtained via Equation (6),

(7) $$L_{s,TCF}=\int_{0}^{2k\pi }\sqrt{\left[{\left[x^{\prime }\left(\theta \right)\right]}^{2}+{\left[y^{\prime }\left(\theta \right)\right]}^{2}+{\left[z^{\prime }\left(\theta \right)\right]}^{2}\right]}d\theta$$

In addition, when the temperature changes, the effective refractive index of the sensing fiber changes due to thermo-optical effects, and the wavelength shift caused by the applied temperature variation can be approximately expressed as [26]:

(8) $$\Delta \lambda_{ij}^{T}=\frac{2}{2n+1}\left[\frac{\partial \Delta n_{eff}^{i-j,s}}{\partial T}L_{s,TCF}+\Delta n_{eff}^{i-j,s}\frac{\partial L_{s,TCF}}{\partial T}+\frac{\partial \Delta n_{eff}^{i-j,h}}{\partial T}L_{h,TCF}+\Delta n_{eff}^{i-j,h}\frac{\partial L_{h,TCF}}{\partial T}\right]\cdot \Delta T$$

## 4. Discussion

Figure 4 presents the schematic experimental setup for measuring the directional torsion of the sensing element. The sensor is clamped between two fiber rotators; the displacement platform is used to control the spacing of the fiber rotators and keep the sensor straight.

To evaluate the effect of the pre-twist angle on the sensor performance, three HTCF structures with pre-twisted angles of 180°, 360°, and 540° were fabricated. The experimental device shown in Figure 4 was used to conduct the torsional measurement of the sensors composed of three HTCF structures. During the experimental demonstration, we use central air conditioning in the clean room to keep the temperature at 23.0 ± 0.1 °C. A table display thermometer with resolution of 0.1 °C was used to monitor the ambient temperature changes in real-time. In order to keep the sensor straight, the sensor is stretched slightly and clamped between two fiber rotators with a distance of 14.1 cm, and the rotatable clamp has a division value of 5°. The rotator on one side is rotated in a CW or CCW direction, while the other is kept fixed. As far as the torsional measurement is concerned, the transmission spectra of the sensor were recorded by rotations of 60 degrees in the clockwise and counterclockwise directions, respectively, with a rotation step of 10 degrees. The technical recorded value was defined as positive in the CW direction and negative in the CCW direction.

**Figure 5:**
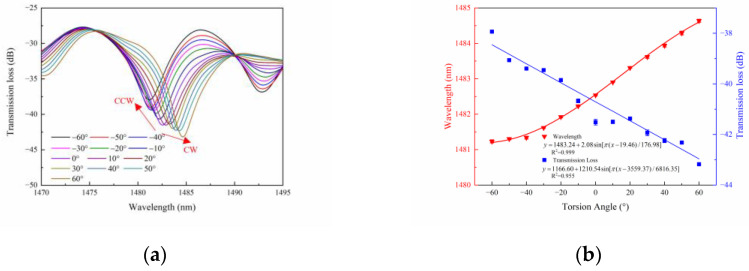

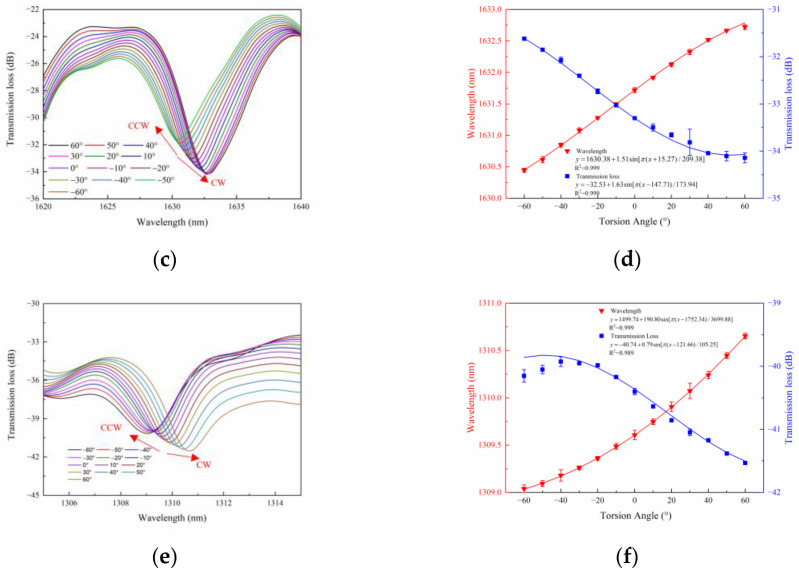
The response and wavelength fitting of the sensor structure about torsion with the pre-twist angles of 180 degrees (**a**,**b**), 360 degrees (**c**,**d**), and 540 degrees (**e**,**f**).

The resonant dips in the interferometric spectrum are monitored, and the data fitting is performed. According to reference [27], the deviation of resonance wavelength conforms to sinusoidal distribution, so sine fitting is used. Figure 5a,c,e highlight the three HTCF structures’ wavelength shift and power change with the angle variation. As is shown in Figure 5, the dip spectrum shifts to a longer wavelength for the CW twist direction and shifts to a shorter wavelength for the twist CCW direction, which is consistent with the previous theoretical analysis. It can be proved that the sensor can discriminate the torsion direction by the change in wavelength.

In general, the maximum wavelength sensitivities are 0.242 nm/(rad/m), 0.161 nm/(rad/m), and 0.105 nm/(rad/m), and the corresponding power sensitivities are −0.04 dB/°, −0.02 dB/° and −0.02 dB/°, as shown in Figure 5b,d,f, respectively. Comparing the experimental results of the three HTCF structures, we can find that the maximum wavelength sensitivity is obtained when the pre-twist angle is 180 degrees. It should be noted that a greater pre-twist angle will result in increased losses. The length of the fiber is fixed at 0.3 cm; the larger the pre-twist angle, the faster the guide mode energy loss in the fiber, resulting in the decrease in interference spectrum energy and the increase in sensor loss, which is not conducive to sensing applications. This can be intuitively seen from Figure 3.

**Figure 6:**
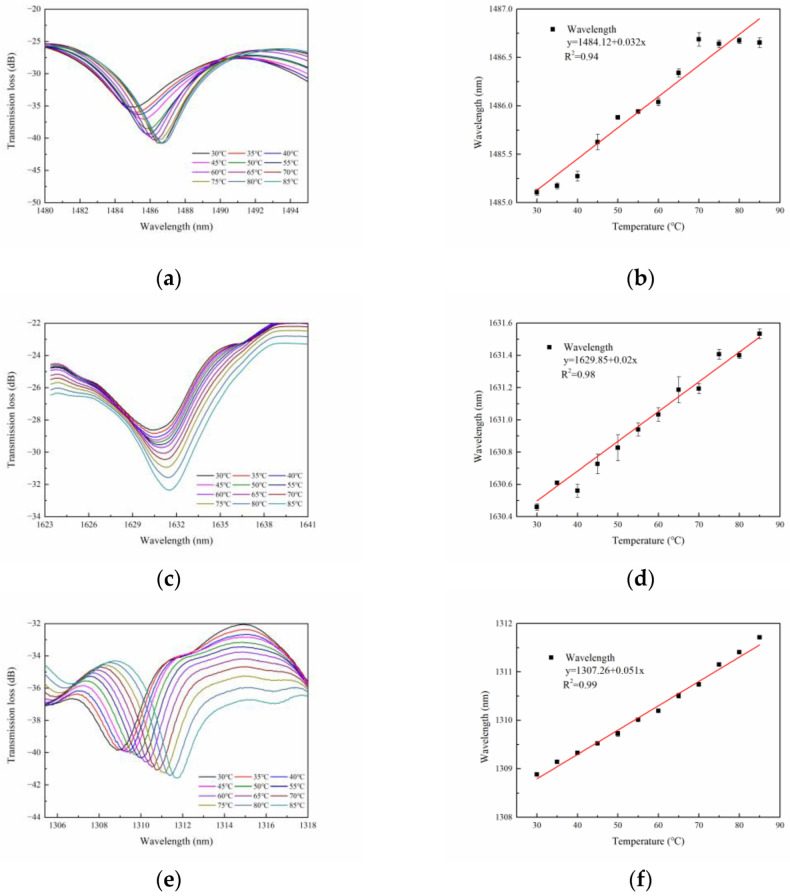
The temperature response of the sensor structure with pre-twisted angles of 180 degrees (**a**,**b**), 360 degrees (**c**,**d**), and 540 degrees (**e**,**f**).

In addition, as temperature crosstalk is a non-negligible factor for optic fiber sensors, we explored the temperature characteristic of the proposed structure. A water bath (HWS-12) with a temperature error of 0.1 °C is used for temperature response. The structure is held in place with composite insulated waterproof tape sticking both ends of the sensor to keep it straight in the water bath. The applied temperature range is from 30 °C to 85 °C with an interval of 5 °C. Considering thermal equilibrium, the transmission spectra were collected when the temperature achieved the target after 30 minutes. In addition, the spectral shift with the temperature is divulged in Figure 6a,c,e, respectively. It is observed that the spectra presents a red-shift behavior as the temperature increases. The wavelength response characteristics of the dips are displayed in Figure 6b,d,f, respectively, where it can be observed that the dips exhibit good linearity as a function of temperature. The measured wavelength sensitivities are 32 pm/°C, 20 pm/°C, and −51 pm/°C, respectively. Therefore, the actual application needs temperature correction or temperature compensation. Table 1 shows the sensing performance comparison between the sensors reported in the literature and the proposed sensor in this work. The proposed sensor has the advantage of simultaneously measuring the torsion angle and torsion direction.

## 5. Conclusions

In conclusion, a highly sensitive directional torsion sensor based on a compact HTCF was demonstrated. The sensor consists of a segment of HTCF and two SMFs, forming an MZI. The HS is implemented by pre-twisting 1 cm long TCF. The performance of the sensor with the pre-twist angles of 180°, 360°, and 540° is experimentally revealed. The results show that the highest twisted sensitivities reach up to 0.242 nm/(rad/m) and −0.04 dB/° respectively when pre-twisted at 180°. Additionally, we calibrate the temperature characteristics of the sensor, while the sensitivity is obtained at 32 pm/°C. The resolution of the sensor is 0.161 nm/(rad/m) and the accuracy is 0.67°. The sensor, with its simplified production process, low cost, compact structure, and high sensitivity, is expected to have potential application prospects in the field where both torsion angle and direction measurements are required.

## Figures and Tables

**Table 1:** Performance comparison of the reported torsion sensors.

Sensor Structure	Sensitivity	Direction Discrimination	Refs.
LPG and TFBG	1.074 dB/(rad/m)	NO	[7]
FM-TFBG and FM-FBG	0.16 dB/°	YES	[28]
PM-PCF	0.014 dB/°	NO	[29]
Square-NCF	1.28615 nm/(rad/m)	NO	[30]
Taper-PMF	2.392 nm/(rad/m)	YES	[13]
PC with PMF	0.29 dB/°	NO	[31]
MMF-SCF-MMF	0.118 nm/(rad/m)	YES	[21]
MMF-SCF-MMF	0.4 nm/(rad/m)	YES	[32]
HCF with Pb	0.1 dB/°	NO	[33]
SMF-HTCF-SMF	0.242 nm/(rad/m) −0.04 dB/°	YES	Our work

## Data Availability

Not applicable.
